# Supplementary figures and images for: Gene-based analyses of the maternal genome implicate maternal effect genes as risk factors for conotruncal heart defects
Source: PLoS One. 2020 Jun 9;15(6):e0234357. doi: 10.1371/journal.pone.0234357 (PMC7282656; doi:10.1371/journal.pone.0234357)

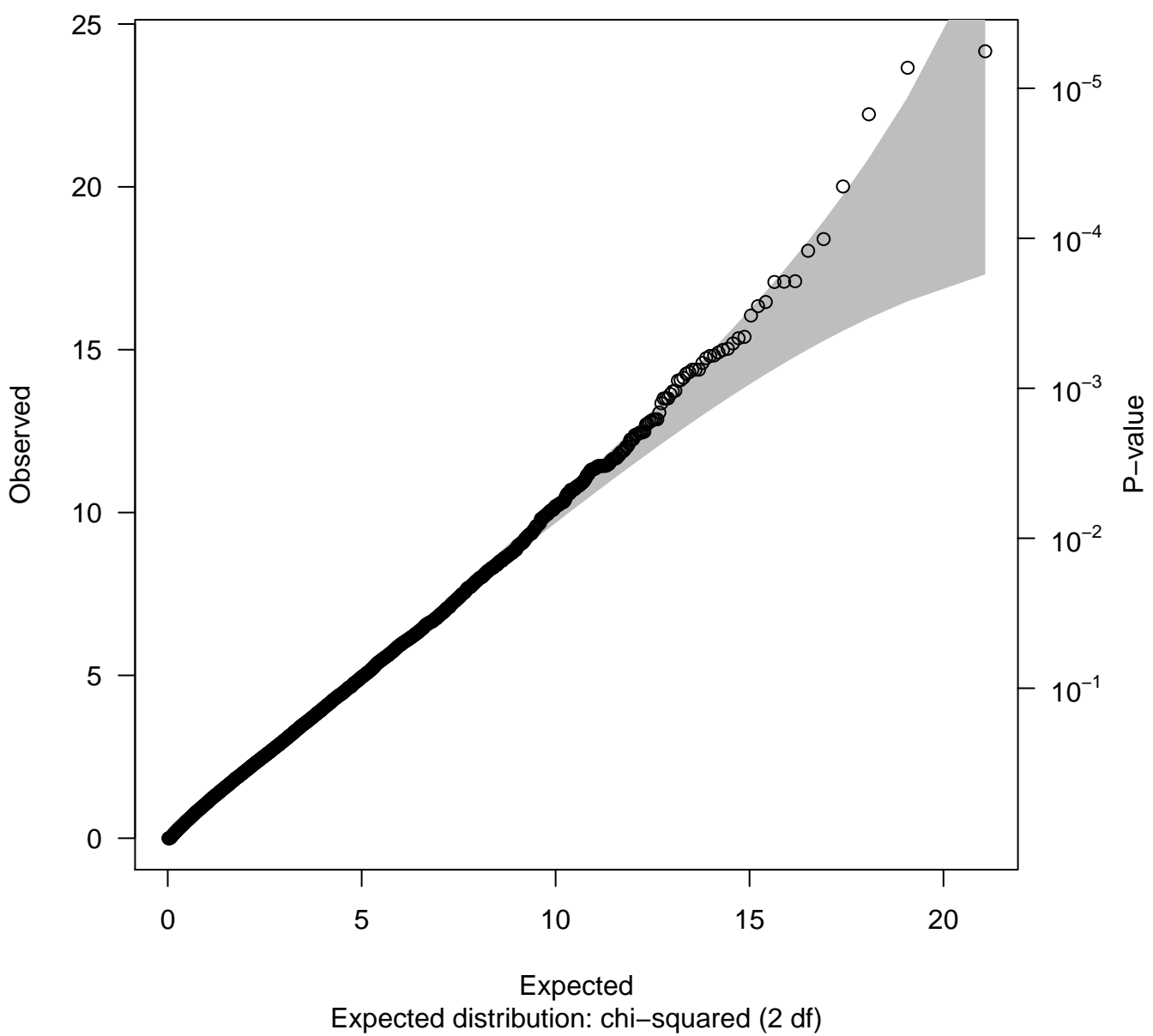

Supplement: S1 Fig — (PDF) [file pone.0234357.s001.pdf]

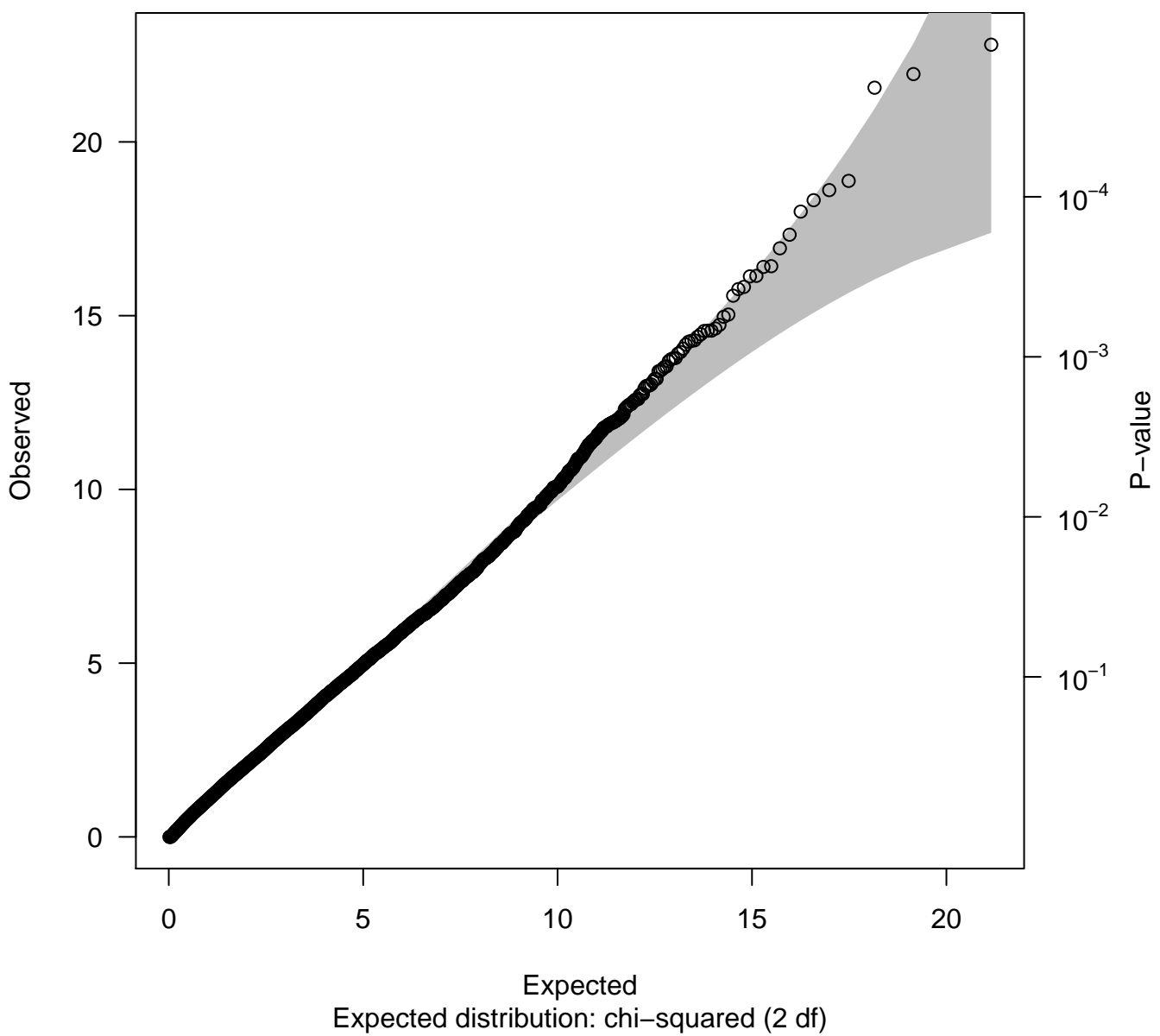

Supplement: S2 Fig — (PDF) [file pone.0234357.s002.pdf]
